# Pulmonary Hyalinizing Granuloma Mimicking Metastatic Lung Cancer

**DOI:** 10.1155/2015/610417

**Published:** 2015-08-05

**Authors:** Nuri Düzgün, Ercan Kurtipek, Hıdır Esme, Meryem İlkay Eren Karanis, İsmet Tolu

**Affiliations:** ^1^Department of Thoracic Surgery, Konya Training and Research Hospital, 42090 Konya, Turkey; ^2^Department of Chest Disease, Konya Training and Research Hospital, 42090 Konya, Turkey; ^3^Department of Pathology, Konya Training and Research Hospital, 42090 Konya, Turkey; ^4^Department of Radiology, Konya Training and Research Hospital, 42090 Konya, Turkey

## Abstract

Pulmonary hyalinizing granuloma is a very rare benign condition, which usually manifests as solitary and sometimes as multiple pulmonary nodules. Deposition of immune complexes in the lung parenchyma due to hypersensitivity reactions is implicated in the etiology of pulmonary hyalinizing granuloma. A 59-year-old female patient who presented to our clinic with complaints of chest pain and cough had bilateral, multiple, and rounded lesions with regular margins suggesting metastatic lung disease. A transthoracic needle biopsy of the nodule was performed in the left pulmonary anterior segment. Biopsy showed no malignancy. Since no diagnosis was made by the biopsy, the patient underwent a video-assisted thoracic surgery. The wedge biopsy reported pulmonary hyalinizing granuloma. We aimed to present the diagnosis and treatment stages of our patient who was diagnosed with pulmonary hyalinizing granuloma in the light of literature review.

## 1. Introduction

Pulmonary hyalinizing granuloma (PHG), which was first described in 1977 by Engleman et al., has been usually reported as individual cases in the world literature [1]. Although its etiology remains unknown, the underlying cause is thought to be deposition of immune complexes in the lung parenchyma which usually occurs following infection or autoimmune process. Cases of PHG with multiple bilateral nodules radiologically mimic metastatic lung carcinoma. The final diagnosis in PHG is established with a histopathological assessment. Patients with solitary PHG nodule have a good prognosis, and they are completely treated with total resection. However, multiple lesions may progress rapidly, leading to extensive involvement.

## 2. Case Report

**Figure 1:**
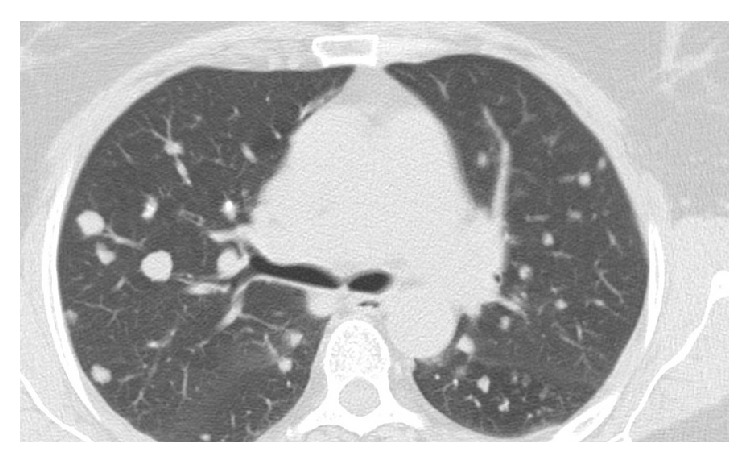
Bilateral multiple nodules with regular margins are detected in the CT of the patient.

**Figure 2:**
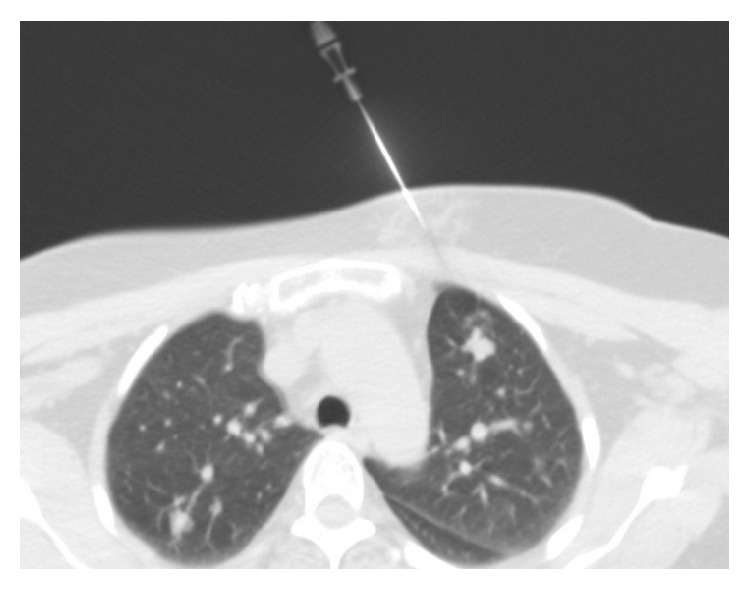
The needle advances toward the nodule in the anterior segment of the left lung during transthoracic biopsy.

**Figure 3:**
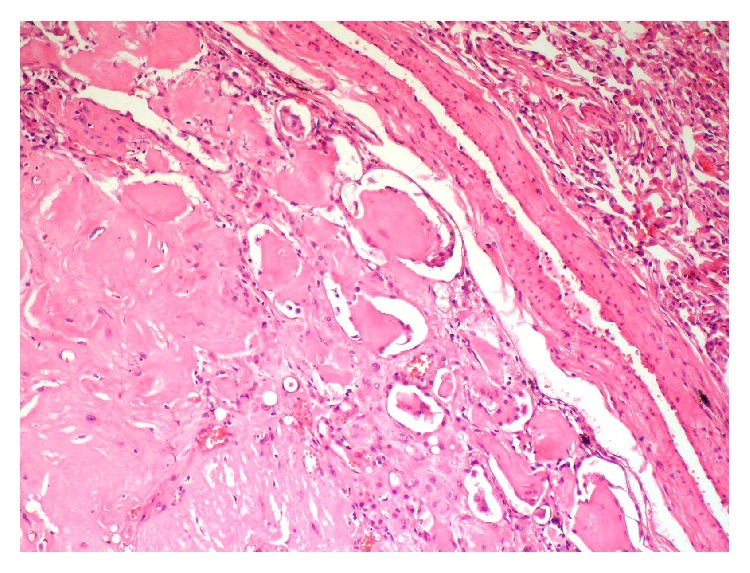
A microscopic view of PHG. A lesion with regular margins containing very hypocellular, keloid-type coarse collagen. HE ×100.

A 59-year-old female patient presented to our clinic with complaints of chest pain and cough. The physical examination and blood tests showed no pathological finding. The patient had no history of tuberculosis or prior lung disease. Additionally, she had well-regulated type 2 diabetes. Computed tomography (CT) showed pulmonary nodules with regular margins and lobulated contours scattered throughout both lungs, the largest measuring 14 × 12 mm in size located in the laterobasal segment of the lower lobe, which suggested metastatic lung disease (Figure 1). Due to suspected malignancy based on these findings, the patient underwent positron emission tomography (PET-CT) both for screening of distant metastasis and for detecting primary tumors. However, there was no significant fluorodeoxyglucose (FDG) uptake in the multiple parenchymal and subpleural nodules. A transthoracic needle biopsy was performed on the anterior segment of the left lung in order to make a diagnosis (Figure 2). Biopsy showed no malignancy. The patient underwent video-assisted thoracoscopic surgery due to lack of diagnosis by biopsy. The shrunken lesion in the posterolateral segment of the right lower lobe was removed by wedge resection. A macroscopic analysis of the wedge resection showed a 1.3 cm rubbery, white, solid mass lesion with regular margins in the cross section. The entire mass was sampled. The cross sections showed a lesion with regular margin containing hypocellular keloid-type coarse collagen areas (Figure 3). There was no atypical epithelial cell, necrosis, and mitosis. Amyloid was not detected with histochemical Crystal Violet and Congo Red stains. PAS staining was performed for differential diagnosis of fungal infections and was found negative. The case was reported as pulmonary hyalinizing granuloma.

## 3. Discussion

PHG is a rare benign lung disease. Generally, regardless of race or gender, the age range of PHG is from 19 to 77 years, with a mean age of 43 years at presentation [2]. Twenty-five percent of patients are asymptomatic. The most common symptoms in symptomatic patients are cough, shortness of breath, and chest pain [1–3]. Our patient also presented with complaints of cough and chest pain, consistent with the symptoms described in the literature. The hyalinizing granuloma is characterized with unilateral and bilateral solitary or multiple nodules which can be radiologically detected, with a diameter ranging from 0.2 to 15 cm (mean 2 cm). The dimensions of the lesions were also consistent with the literature in our patient. The regular margins suggested metastatic lung carcinoma. Similarly, in a case report by Unlu et al., the patient with PHG had a radiological appearance of metastatic lung cancer [4]. For radiological differential diagnosis, sarcoidosis, rheumatoid nodules, Wegener's granulomatosis, tuberculosis, and amyloidosis as well as primary or metastatic tumors of the lung should be considered. Our patient had no history of tuberculosis or prior lung disease. TFNAB, endobronchial sampling, biopsies, and bronchoalveolar brushing and lavage are often not efficient for diagnosis [5]. Moreover, pulmonary hyalinizing granuloma can be confused with nodular amyloidosis, fungal infections, and inflammatory myofibroblastic tumors. Inflammatory myofibroblastic tumors are more cellular and consist of inflammatory cells such as lymphocytes, histiocytes, eosinophils, and leucocytes [6]. However, pulmonary hyalinizing granulomas are more hypocellular and have rough collagen such as keloid and sparse lymphocytes [7]. Differentiation from malignancy and final diagnosis usually require surgical biopsy. Surgical procedure can be performed for diagnostic purposes in patients with bilateral or multiple nodules as well as for complete resection in patients with solitary lesions [8]. The final diagnosis is made based on the histopathological analysis of the sample. Patients with solitary nodule have a good prognosis, and they are completely treated with total resection. Although PHGs typically have slow growth, they may show a rapid growth in the presence of multiple lesions. There are some publications recommending addition of glucocorticoids to the therapy although their effect remains unclear [9, 10]. Our patient was relieved after initiation of steroid therapy upon diagnosis.

In conclusion, PHG can be misdiagnosed as several benign and malignant diseases. Therefore, pulmonary hyalinizing granuloma should be considered in differential diagnosis of lesions suggesting metastatic lung carcinoma, particularly without any primary focus as in our case.
